# Six Weeks of Basketball Combined With Mathematics in Physical Education Classes Can Improve Children's Motivation for Mathematics

**DOI:** 10.3389/fpsyg.2021.636578

**Published:** 2021-03-26

**Authors:** Jacob Wienecke, Jesper Hauge, Glen Nielsen, Kristian Mouritzen, Linn Damsgaard

**Affiliations:** Department of Nutrition, Exercise and Sports, University of Copenhagen, Copenhagen, Denmark

**Keywords:** motor-enriched learning, motivation, academic learning, children, intrinsic motivation, classroom-based mathematic, embodied cognition

## Abstract

This study investigated whether 6 weeks of basketball combined with mathematics once a week in physical education lessons could improve children's motivation for mathematics. Seven hundred fifty-seven children (mean age = 10.4 years, age range: 7–12 years) were randomly selected to have either basketball combined with mathematics once a week (BM) or to have basketball sessions without mathematics (CON). Children in BM and CON motivation for classroom-based mathematics were measured using the Academic Self-Regulation Questionnaire (SRQ-A) before (T0) and after the intervention (T1). Among the BM, levels of intrinsic motivation, feelings of competence, and autonomy were measured using the Post-Experimental Intrinsic Motivation Inventory (IMI) questionnaire acutely after a basketball session combined with mathematics and immediately after a session of classroom-based mathematics. BM had significantly higher acute levels of perceived autonomy (+14.24%, *p* < 0.0001), competencies (+6.33%, *p* < 0.0001), and intrinsic motivation (+16.09%, *p* < 0.0001) during basketball sessions combined with mathematics compared to when having classroom-based mathematics. A significant decrease in the mean for intrinsic motivation was observed from T0 to T1 for CON (−9.38%, *p* < 0.001), but not for BM (−0.39%, *p* = 0.98). BM had a more positive development in intrinsic motivation compared to CON from T0 to T1 (*p* = 0.006), meaning that BM had a positive influence on children's intrinsic motivation for classroom-based mathematics. This study indicates that basketball combined with mathematics is an intrinsically motivating way to practice mathematics, which also has a positive influence on children's general intrinsic motivation for mathematics in the classroom.

## Introduction

Children's capacity to formulate, employ, and interpret mathematics, *mathematical literacy*, plays a central role in making well-founded judgments and decisions in life (Grinstein and Lipsey, 2001). Over the past decades, it has become more common to explore different learning approaches to stimulate children's mathematical learning to optimize the educational programs within mathematical subjects. Especially, intervention studies with focus on using physical activity to improve cognitive and academic performance have received a lot of attention (Sibley and Etnier, 2003; Best and Miller, 2010; Donnelly et al., 2016; Mavilidi et al., 2020).

Less focus has been aimed at the potentials of integrating physically active learning activities into the learning activities (Diamond, 2015; Pesce et al., 2016). The main purpose of the present study was to develop a model, which combines physical activity and mathematics in a play-based setting, and investigate how this play-based model affects children's motivation for mathematics.

An important contributor to academic achievement is motivation, which plays a central role in learning mathematics (Singh et al., 2002). Wienecke and Damsgaard (2020) describe how math combined with elements of basketball can be used as a practical model of play-based physical activity which can create a dynamic and enjoyable learning environment (Wienecke and Damsgaard, 2020). Valentini and Rudisill (2004) emphasize the possibilities of using basketball as an inclusive learning setting (Valentini and Rudisill, 2004). Mavilidi et al. (2018) used elements of basketball to support and reinforce the learning of English language concepts. The study indicates that children's joy and positive feelings rate high when having this learning setting (Mavilidi et al., 2018). Despite the promising data, no other previous study has focused on motivation and basketball combined with mathematics. Therefore, the present study aims to investigate how combining basketball with mathematics in a school setting affects children's motivation for mathematics.

Motivation that facilitates academic achievement can be facilitated by a positive learning environment and positive experiences with the academic task (Fortier et al., 1995; Singh et al., 2002). However, with age, students' reporting of enjoyable and stimulating school days decreases, especially between ages 11–15 (Gutman et al., 2010; Rasmussen et al., 2014).

According to the Self Determination Theory (SDT), there are different types of motivation with different levels of autonomy and different effects on academic achievement and development (Ryan and Deci, 2000). SDT distinguishes between four types of extrinsic motivation with various levels of external control and autonomy. These are: external regulation, where behavior is motivated by avoiding punishment; introjected regulation, where involvement is regulated by ego inflation through attaining success relative to others' expectations; identified regulation, where the individual recognizes and identifies with the value of the behavior; and integrated regulation, where the underlying value of an activity is not only recognized, but is also in coherence with other parts of the individual's deeper value system and identity (Deci and Ryan, 1985; Reis et al., 2000). Intrinsic motivation, on the other hand, is the behavior driven by reasons that are ingrained in the activity itself. Intrinsic motivation is the most autonomous type of motivation, and is a drive to do something because it is enjoyable and interesting more than doing something for extrinsic reasons and benefits. To experience competence, autonomy and relatedness are considered basic psychological needs, which must be fulfilled when doing an activity for sustaining the intrinsic motivation for that activity (Deci et al., 1999).

The more autonomous types of motivation (Intrinsic Motivation, Integrated and Identified Regulation) have shown to be positively related to long-term involvement in learning activities, and higher school achievement, better understanding of taught concepts, improved school satisfaction, and a lower school dropout rate (Gottfried, 1985; Ryan and Deci, 2002; Ryan, 2009; Gutman et al., 2010). Whereas, more controlled forms of motivation have been associated with students' experience of distraction, negative feelings, and lower grades (Guay et al., 2010).

Middleton showed that the more interest a student has in mathematics, the more effort the student is willing to put in, the more the student experienced the activity as enjoyable, and the more they are willing to persist in the face of difficulties (Middleton, 1995). It is likely that integrating concrete, meaningful, and purposeful physical activities such as basketball in the teaching and practicing of mathematics supports children's need for feeling autonomous and competent more than the traditional classroom teaching, and is therefore, more intrinsically motivating. The feeling of autonomy may be fostered through higher perceived purposefulness of the activities and mathematics. As such, the feeling of competence may be promoted by the involvement of less abstract and more hands-on skills and learning approaches. It is also likely that using mathematics to solve concrete tasks, as in the basketball exercises in this study, may help students to recognize and identify with the value of practicing and learning mathematics, i.e., promote the extrinsic motivation for mathematics in terms of identified regulation.

It is shown that participating in physical activity has significant benefits for children's cognition and academic education both with single bouts of physical activity (Ferris et al., 2007; Skriver et al., 2014; Hillman et al., 2019), regular physical activity (Broussard, 2004; Geertsen et al., 2016; Damsgaard et al., 2020), and high physical activity levels (Hillman et al., 2014; Donnelly et al., 2016; Marques et al., 2018).

Also, motor-enriched learning, where learning of a subject is combined with meaningful motor activities, has shown a positive effect on academic content (Beck et al., 2016). Damsgaard et al. (2020) found that motor-enriched learning improved children's academic learning (letter recognition), and the children who performed motor-enriched learning had a higher intrinsic motivation for the academic content. Teaching situations where physical activity are integrated meaningfully, may therefore influence both children's motivation and there academic performance in a positive way (Broussard, 2004; Geertsen et al., 2016; Damsgaard et al., 2020).

Our aim, with integrating mathematical tasks with concrete and physically active basketball tasks, was to make the learning activities more interesting, meaningful, play-based, and fun for the children. More specifically, we hypothesize that basketball combined with mathematics is a concrete physically active way of employing, practicing, and learning mathematics that will result in students feeling a higher degree of autonomy and competence, and is more intrinsically motivating than classroom-based mathematics.

Based on the hierarchical nature of motivation (Fortier et al., 1995; Vallerand, 2000), we further hypothesize that these positive situational experiences with mathematics will have a positive impact on the children's motivation for mathematics in general, and also in the classroom. As the basketball sessions combined with mathematical activities do not involve more cooperation and group work, we do not expect that it will have any effects on experiencing relatedness.

## Materials and Methods

### Participants

**Figure 1:**
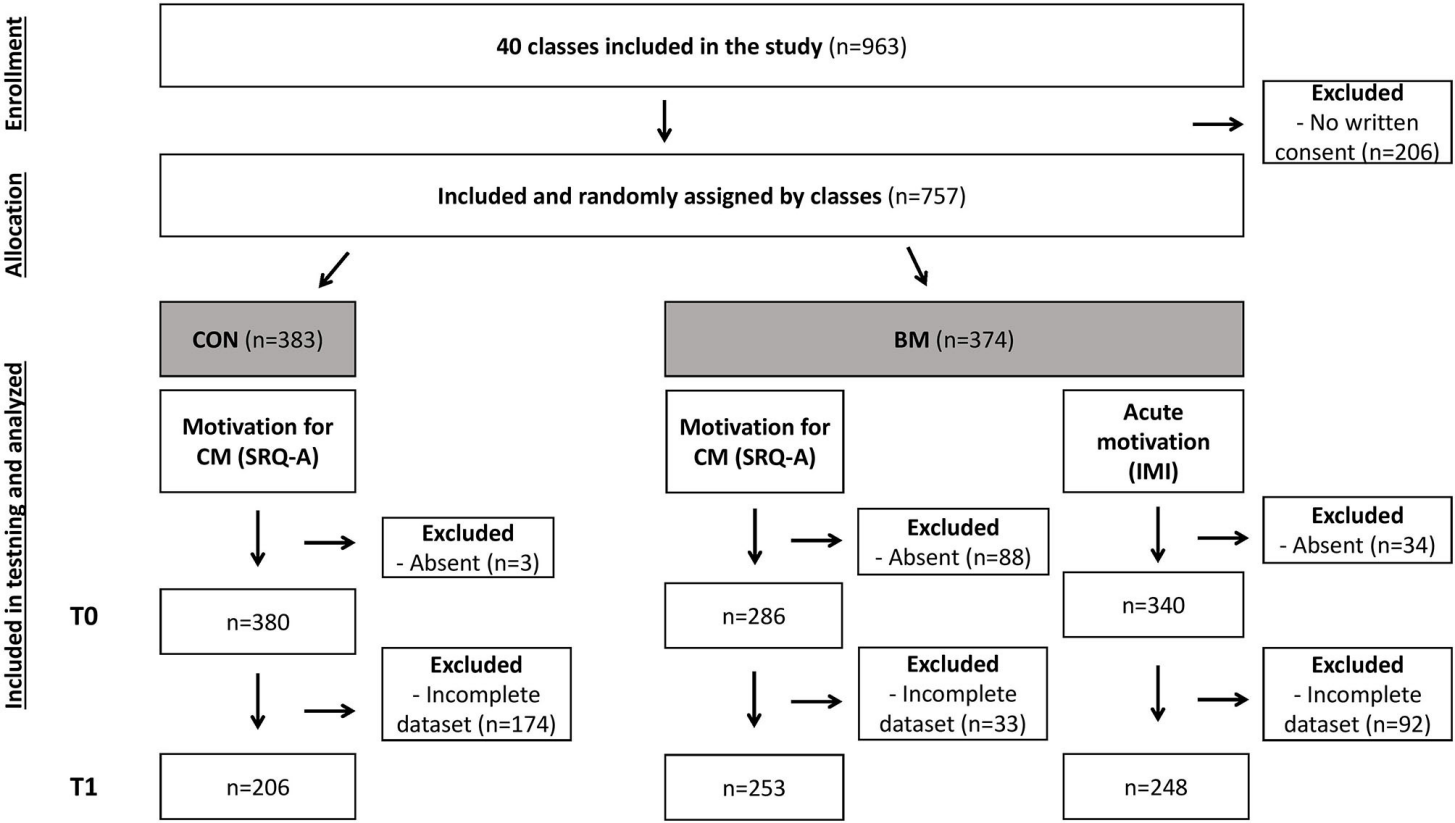
Flow diagram. Flow diagram of the study. Seven hundred fifty-seven children were randomly assigned to either basketball sessions without mathematics (CON) or basketball sessions combined with mathematics (BM). The children performed a questionnaire (SRQ-A) about their motivation for classroom-based mathematics (CM) before (T0) and after an intervention period of 6 weeks (T1). BM also performed a motivation questionnaire (IMI) acutely after a basketball session combined with mathematics and immediately after a session of classroom-based mathematics. Only complete cases were analyzed.

In total, 757 students took part in this school-based study after obtaining written consent from parents, corresponding to 78.60% of the invited children. All students were recruited from five Danish elementary schools from within and outside the Copenhagen area. The included children came from 40 different classes at different grade levels; elementary school (1st to 3rd grade) and middle school (4th to 5th grade) (see Table 1). 125 participants were absent at tests days and were excluded. 207 participants were excluded from the analysis of the Academic Self-Regulation Questionnaire (SRQ-A) and 92 participants from the analysis of the Post-Experimental Intrinsic Motivation Inventory (IMI) due to incomplete data (see flow diagram, Figure 1). In total, the statistical analysis was made upon 459 participants for the SRQ-A questionnaire and 248 for the IMI questionnaire. At every school, the classes were randomly selected to have either BM or CON with just regular basketball training during their weekly PE lesson. The study was approved by the local Ethical Committee at the University of Copenhagen, Denmark (protocol: 504-0016/17-5000) and was carried out in accordance with the Helsinki Declaration II.

**Table 1:** Demographics for the two intervention groups (CON, BM).

	**CON**			**BM**		
**Grade level**	**Total**	**ES**	**MS**	**Total**	**ES**	**MS**
Participants (n)	206	105	101	253 (248)	107 (135)	146 (113)
Age (Years)	10.40 ± 0.42	9.40 ± 0.44	11.40 ± 0.40	10.33 ± 0.39	9.30 ± 0.39	11.35 ± 0.38
Sex (% Boys)	50	51	49	54 (52)	49 (52)	58 (42)

Data reported as mean ± SD. CON, Control Group (Basketball sessions without mathematics); BM, Basketball sessions combined with Mathematics; ES, Elementary School; MS, Middle School. Baseline data made upon the included data from the SRQ-A data. SRQ-A data is based on the pre and post measurements. Note that the numbers represented in parentheses indicate the included participants in the IMI data analysis within the BM group, which is an acute measure.

### Intervention

**Figure 2:**
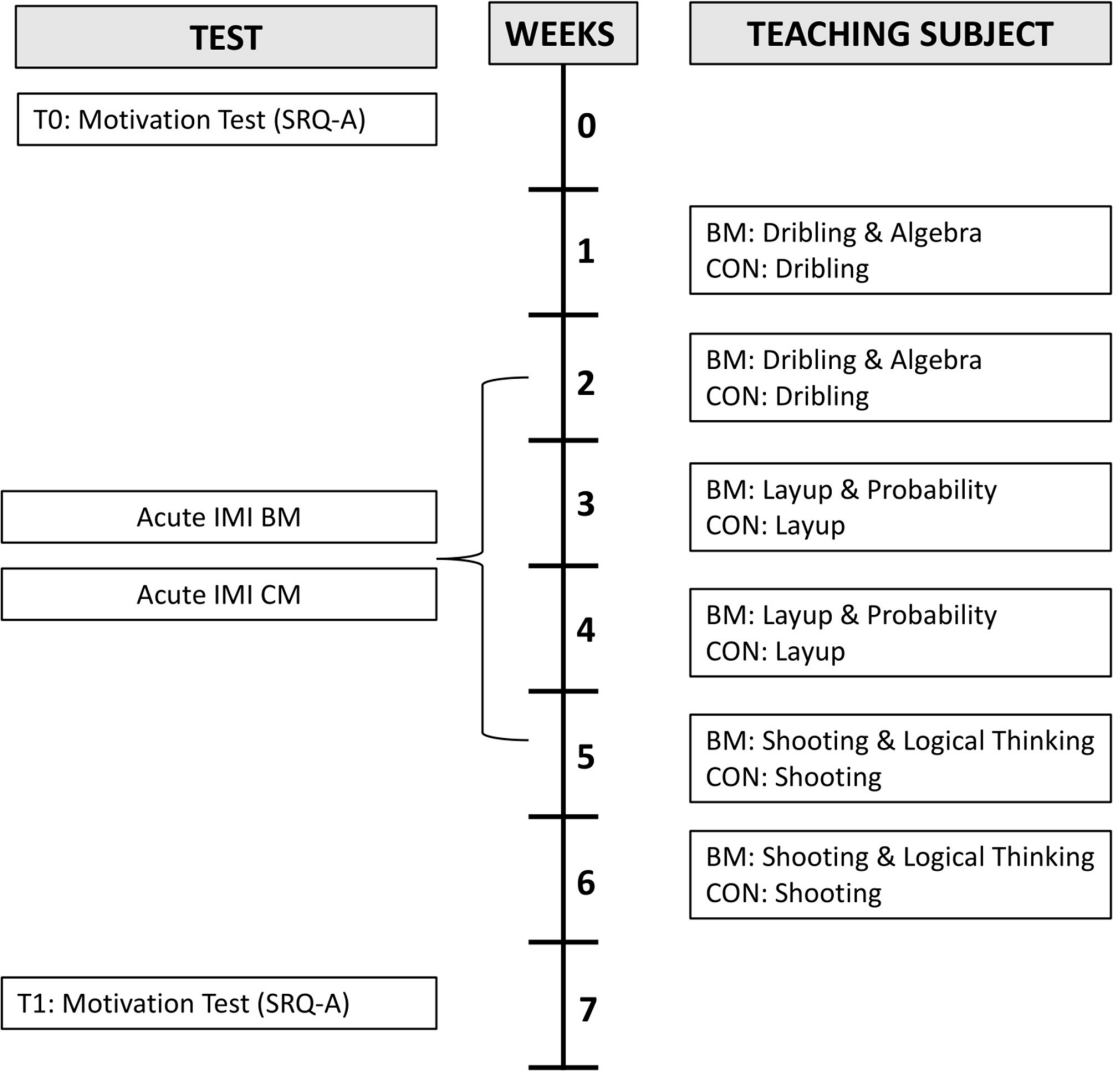
Intervention overview. Two groups (BM: Basketball combined with Mathematics and CON: Basketball without mathematics) were tested before (T0) and after (T1) an intervention period of 6 weeks with an SRQ-A motivation questionnaire. Within the BM-group, the children performed acutely motivation questionnaire (IMI) in basketball sessions combined with mathematics (Acute BM), and in classroom-based sessions with mathematics (Acute CM) randomly in week 2–5. The figure also shows the different teaching themes in CON and BM.

The 374 students in the intervention group received teaching in basketball combined with mathematics built into the exercises (BM) once a week over a coherent 6 week period. Each lesson had a duration of 90 min including time to change into sportswear. That allowed ~70 min of isolated time to focus on the exercises with basketball combined with mathematics. The control group (CON), which consisted of 383 students, received the same amount of teaching in basketball without any kind of mathematics built into the exercises. In both groups (CON and BM), the lessons were planned and ran by external trained researchers, specially recruited and educated for this project, but supported by and in cooperation with the class' normal teacher. The content of mathematics in the intervention group (BM) was adjusted to fit the actual level of each participating class by consulting their mathematics teacher and in compliance with the national curriculum. Each teaching lesson had the following structure: introduction, a warm-up-activity, exercise 1, exercise 2, exercise 3, and in the end, a finishing exercise where the teachers summarized the lesson's theme and activities with the children. During the lesson (in between the exercises), the teachers had a dialog about the elements in the practice both in relation to the task, basketball skills, and mathematics (only in the BM group). The mathematical theme was meaningfully built into the exercises. The children, for example, were tasked with collecting and calculating mathematical information through different basketball exercises. Other exercises were more play-based, where the children had to perform mathematics (multiplication, addition, etc.) and basketball skills to win games. For a more detailed overview of mathematical content and basketball skills, see Figure 2 and the website: www.basketballmathematics.org. The design setup was a mix between different exercises, where some exercises were more motor-enriched and some were more focussing on high loaded cardiovascular.

In structuring the transitions between the lessons, transfer and recognition were key elements. Recognition refers to how the children should easily recognize the game and the structure to reduce misunderstandings and confusion, and therefore, specific exercises were reused. Transfer refers to the aspect of reusing exercises and lesson structure, but introducing a shift in the content, for example, from lay-up to shooting from a distance. Similarly, the mathematics could change from addition to subtraction. From a pedagogical perspective, the reuse of games made it easy to change the content of the exercises slightly and still keep the children's attention, and thereby, support them in understanding the structure of the exercise.

### Test Procedures

Data about age and sex were collected prior to the baseline measures for all children. For the BM group, measures of acute intrinsic motivation (IMI) and the satisfaction of the basic psychological need for autonomy and competence during the two ways of teaching mathematics were collected immediately after one lesson with basketball combined with mathematics, and immediately after one lecture of classroom-based mathematics (CM). For the two groups (CON, BM), measures of the children's motivation for CM were collected at baseline T0 and were post-evaluated after the 6 weeks of intervention period (T2).

## Measures

### Post-Experimental Intrinsic Motivation Inventory (IMI)

The Post-Experimental Intrinsic Motivation Inventory (IMI) (McAuley et al., 1989) was used to measure acute intrinsic motivation and experiences of competence and autonomy for the BM group. All participating school classes in BM completed the questionnaire in lessons 2–5 at the end of a normal CM lesson during the intervention (see Figure 2), and also, after one session of basketball combined with mathematics.

IMI measures participants' subjective experience while performing an activity in an experiment, and has been found to be a valid self-reported measure of intrinsic motivation (McAuley et al., 1989; Markland and Hardy, 1997). The scale has previously been used in other studies on the motivational effect of integrating physical and learning activities in primary schools (Vazou et al., 2012).

As a measure of intrinsic motivation, four items from the Interest/Enjoyment scale were used. The used items were: “I really enjoyed the activities”; “The activities were fun”; “I thought it was boring” [Reverse question (R)]; “I thought the activities were interesting.”

To measure the children's experience of autonomy, three items from the Perceived Choice subscale were used: “I believe I had some choice about doing this activity”; “I did this activity because I wanted to”; “I did this activity because I had to” (R).

To measure the children's experience of competence, the following items from the Perceived Competence subscale were used; “I think I am pretty good at this activity”; “I am satisfied with my performance at this task”; “This was an activity that I couldn't do very well” (R).

Items were translated into Danish using a translation-backtranslation process (Streiner et al., 2015). Because the scales were used on children, the original 7-point response-scale was converted to a 4-point scale (1; not true at all, 2; Only slightly true, 3; almost true, 4: True). The investigator read the questions aloud for the children one by one. As an introduction to the specific questions, children were asked how they experienced the lesson they had just taken.

### Motivation for Classroom-Mathematics/CM (SRQ-A)

For all participating school classes in both CON and BM, children's motivation for mathematics was measured using the Academic Self-Regulation Questionnaire (SRQ-A). The questionnaire was filled in during a CM lesson 1 week before the 6 weeks intervention (T0) and 1 week after the last lesson of the intervention period (T1). The questionnaire was completed together with the children's own mathematics teacher.

The SRQ-A is a widely used SDT-based domain-specific 32-item self-report instrument, developed for measuring the degree of different types of motivation for doing schoolwork among children in the late primary and lower secondary school. The SRQ-A uses scales to measure both extrinsic types of motivation (external regulation, introjected regulation, and identified regulation) and intrinsic motivation. Items are organized in four main topics: (1) “Why do I do my homework?,” (2) “Why do I work on my classwork?,” (3) “Why do I try to answer hard questions in class?,” and (4) “Why do I try to do well in mathematics?.” In the present study, the homework domain was not included, as regular homework is not common at the Danish grade levels presently studied, resulting in a questionnaire of 24 items.

The questions are answered on a four-point Likert scale (1 = Not at all true, 2 = Not very true, 3 = Sort of true, and 4 = Very true) and summed up to calculate scores for the five types of motivation across the four areas. The SRQ-A was originally validated by Ryan and Connell (1989) for students in Grades 3–6 (approximate age: 8–12). The SRQ-A is widely used and has shown across studies, samples, and contexts to be a moderately reliable measure, which is stable across subscales (Burton et al., 2006; Dettweiler et al., 2015). Following a translation/back-translation process (Streiner et al., 2014), the instrument was translated from English into Danish.

### Statistical Analysis

#### Psychometric Qualities of the Included Measures

The validity of the SRQ-A and IMI scales were estimated by conducting two exploratory structural equation modeling (ESEM) analysis adjusting for clustering on grade level and school, in Mplus (Muthén and Muthén, 2012). For IMI, measures collected from the intervention group at BM and CM were evaluated. For SRQ-A baseline, measures for the total sample were evaluated. Loadings and cross loadings and model fit were inspected. Loadings >0.3 on intended factor and < 0.3 on unintended factors were considered acceptable. Criteria used to indicate a good model fit were: Chi2/df < 5.00, CFI > 0.95, TLI > 0.95, RMSEA < 0.06 (Hu and Bentler, 1999). To estimate the internal reliability of the psychometric scales, Cronbach's alpha values were calculated with values above 0.6 indicating an acceptable internal consistency of the items (Ponterotto and Ruckdeschel, 2007).

#### Analysis of Intervention Efficacy

The statistical analyses of intervention efficacy were performed in R Studio (R Core Team, Vienna, Austria).

Data from the acute motivation questionnaire (IMI) were analyzed for the intervention group using paired *t*-test to identify possible differences in means.

Data from the SRQ-A questionnaire were analyzed using a linear mixed model with group-time interaction as fixed effects, using the R-packages lme4 (Bates et al., 2017). The data was analyzed for group x time interactions with CON and BM as groups and time were T0 and T1. To account for the cluster structure and the repeated measures in the data, “subjects,” “school,” and “grade-level” were added as fixed effects. Ratio Tests were used to reveal group x time interactions effects for differences before and after the 6 weeks of intervention. Subsequently, if the test for interaction was significant, pairwise comparisons between delta values were used to characterize the interaction effect. To reduce the problem of multiple testing, only relevant model-based specified comparisons were performed including the comparisons of interest (time and group differences) using the *emmeans* R-package (https://CRAN.R-project.org/package=emmeans).

The linear mixed model was chosen as a statistical tool, due to the possibilities in the model when working with repeated observations. The model can be used when dealing with missing data, treating continuous and categorical responses as well as unprincipled methods of modeling heteroskedasticity and non-spherical error variance (for either participant or item) (Baayen et al., 2008). The linear mixed effect models have addressed each of these concerns, and offer, thereby, a better approach than univariate ANOVA.

For all tests, a significance level of 0.05 was applied. Data are reported as means ± SD unless otherwise stated.

## Results

### Psychometric Properties of the Motivation Measures

For measures taken within the intervention group for Acute Intrinsic Motivation (IMI) at both Basketball combined with mathematics (BM) and Classroom-based mathematics (CM), ESEM analysis of the IMI with distinct factors for perceived autonomy, competence, and intrinsic motivation showed acceptable high loadings on the intended factors and no issues with cross loading. The model fit met the criteria for a good model fit. For BM, RMSEA = 0.070 (90% CI 0.041–0.100); CFI = 0.985; TLI = 0.963. For CM, RMSEA = 0.028 (90% CI 0.001–0.066); CFI = 0.999 and TLI = 0.996. Results depicting the factor loadings of items of the IMI can be seen in Appendix 1.

ESEM analysis of the SRQ-A showed some minor issues with one low item loading in identified regulation and one low item loading on external regulation as well as some cross loading from external regulation to introjected regulation. However, the model fit indices met criteria for a good model fit [RMSEA = 0.048 (90% CI 0.041–0.055); CFI = 0.933; TLI = 0.900]. It was decided to use the original factor structure in the further analysis in this present study since the original factor structure has been validated and used in many studies, the model fit was good and finally, that the Cronbach's alpha values were acceptable. Results depicting the factor loadings of items of the SRQ-A can be seen in Appendix 2.

Both the SRQ-a scales and the IMI scales all had Cronbach's alpha values above 0.6.

### Acute Intrinsic Motivation (IMI) Within BM

**Figure 3:**
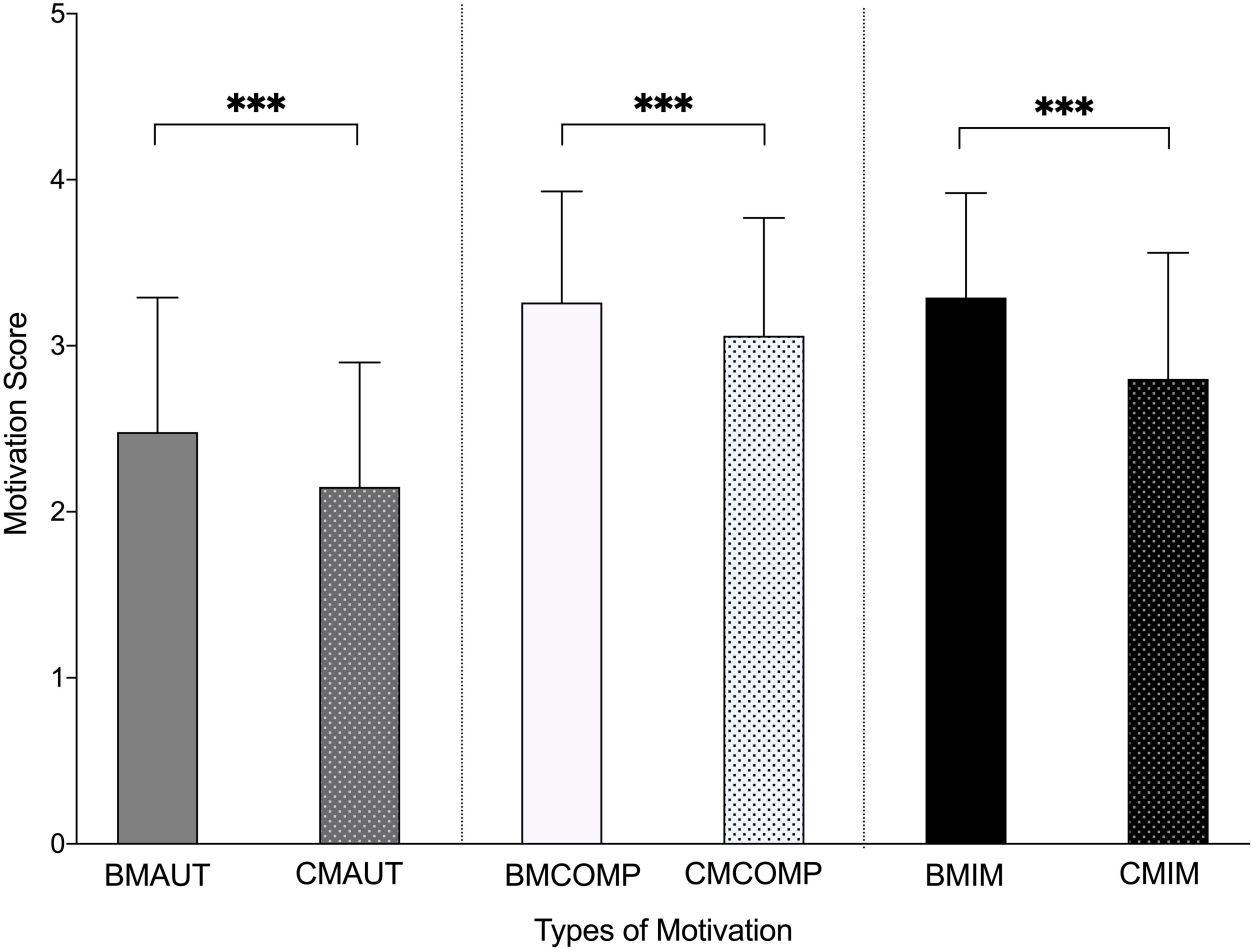
Acutely Motivation (IMI) for the Intervention Group (BM). Acutely IMI measures for BM (Basketball sessions combined with mathematics). Motivation for sessions with basketball combined with mathematics (BM) were compared with motivation for classroom-based mathematics (CM) for the three different motivation types; autonomy (AUT), competencies (COMP), and intrinsic motivation (IM), *Indicates a significant difference between the two teaching methods (basketball combined with mathematics and classroom-based mathematics).

The children in the intervention group participating in basketball sessions combined with mathematics (BM) had significantly higher acute levels of perceived autonomy (+14.24%, *p* < 0.0001), competencies (+6.33%, *p* < 0.0001), and intrinsic motivation (+16.09%, *p* < 0.0001) during basketball sessions combined with mathematics compared to when having classroom mathematics (CM), see Figure 3, Table 2.

**Table 2:** Acute Motivation (IMI) for the intervention group, BM, for sessions with Basketball combined with Mathematics (BM), and for Classroom-based Mathematics (CM).

**Motivation measures**	**BMAUT**	**CMAUT**	**% DIFF**	**BMCOMP**	**CMCOMP**	**% DIFF**	**BMIM**	**CMIM**	**% DIFF**
BM (*n* = 248)	2.48 ± 0.81	2.15 ± 0.75^*^	+14.25	3.26 ± 0.67	3.06 ± 0.71^*^	+6.33	3.29 ± 0.63	2.80 ± 0.76^*^	+16.09

*Data reported as mean ± SD and percentage difference (% diff). Motivation measures are reported as BMAUT (Motivational autonomy for Basketball sessions combined with Mathematics/BM), BMCOMP (Motivational competencies for BM), BMIM (Intrinsic motivation for BM), CMAUT (Motivational autonomy for Classroom-based Mathematics/CM), CMCOMP (Motivational competencies for CM), CMIM (Intrinsic motivation for CM)*.

* *Indicates a significant difference between the same motivation factor, but in different teaching approaches (BM and CM)*.

Subgroup analyses based on grade level showed significantly higher levels for both elementary school (ES) and middle school (MS) for basketball sessions combined with mathematics compared to CM in perceived autonomy (ES: +11.67%, *p* = 0.002; MS: +14.51%, *p* < 0.0001) and intrinsic motivation (ES: +13.23%, *p* < 0.0001; MS: +17.12%, *p* < 0.0001). However, only children in middle school experienced significantly higher levels of competencies (+17.12%, *p* < 0.0001). No other significant differences were found between age groups, sex, or other subgroups.

### Motivation for Classroom-Based Mathematics (SRQ-A)

**Figure 4:**
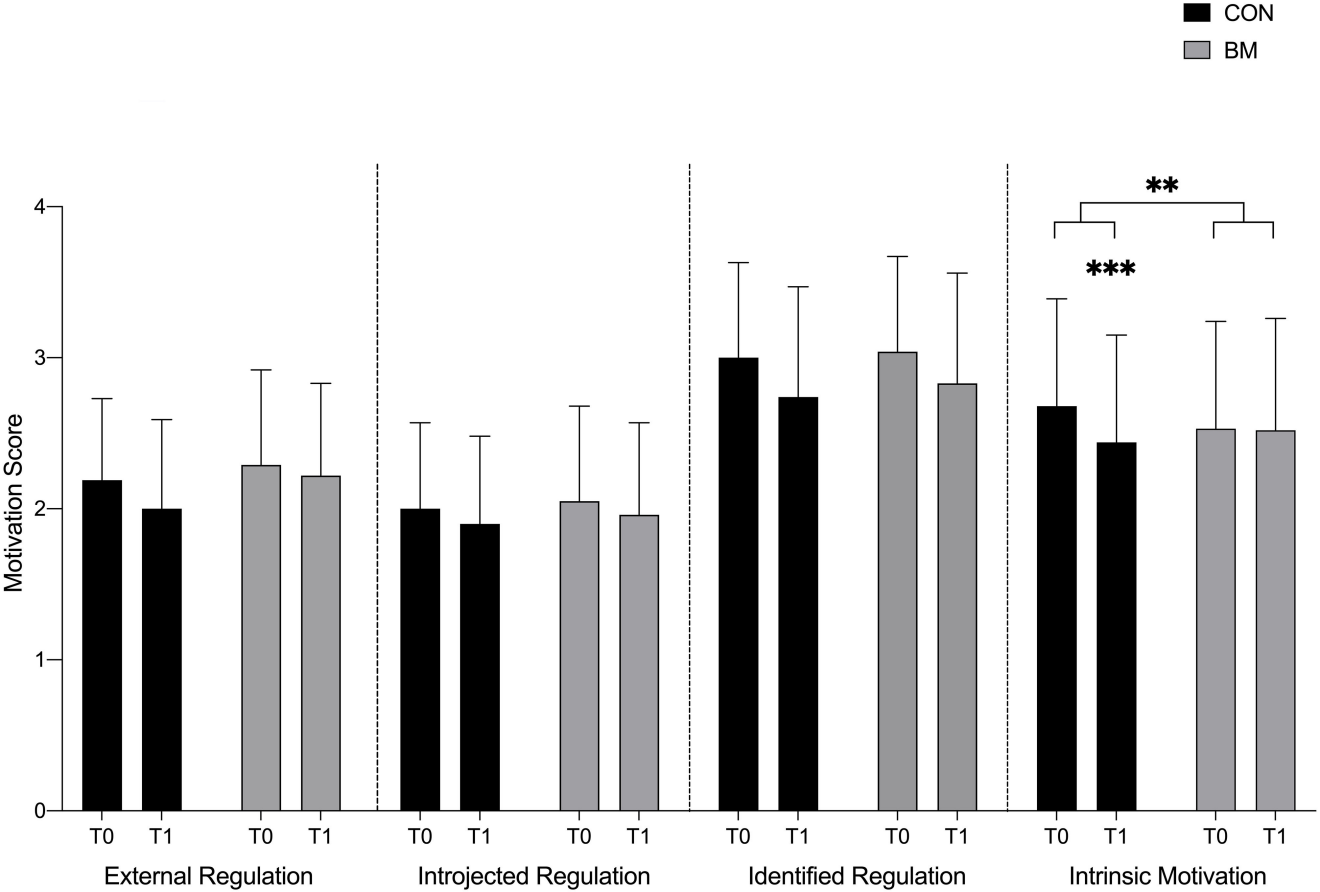
Motivation (SRQ-A) for Classroom-based Mathematics for the two Intervention Groups (CON, BM) at T0 and T1. Motivation for classroom-based mathematics before (T0) the 6 weeks intervention period and after (T1) for the four motivation types; External Regulation, Introjected Regulation, Identified Regulation, and Intrinsic Motivation. A significant decrease in intrinsic motivation (*p* < 0.001, ***) was seen from T0 to T1 for CON, and a significant interaction was found for BM compared to CON from T0–T1 (*p* = 0.006, **) for intrinsic motivation.

Likelihood Ratio Test showed a global significant interaction between time and group (*p* = 0.002) for intrinsic motivation for mathematics. Further analyses showed a significant decrease in means of intrinsic motivation from T0 to T1 for CON [-9.38%, *p* < 0.001 but not for BM (+0.39%, *p* = 0.98)], see Figure 4, Table 3. A significant interaction was found for BM compared to CON from T0–T1 (*p* = 0.006) for intrinsic motivation, meaning that BM had a more positive influence on children's intrinsic motivation for classroom-based mathematics compared to CON. No differences were seen between the intervention and control groups in changes in any of the extrinsic types of motivation for mathematics, where both groups showed insignificant declines.

**Table 3:** Motivation score from SRQ-A questionnaire from T0 and T1 for classroom-based mathematics for the two intervention groups (CON and BM).

**Motivation measures**	**External regulation**			**Introjected regulation**			**Identified regulation**			**Intrinsic motivation**		
	**T0**	**T1**	**% Diff**	**T0**	**T1**	**% Diff**	**T0**	**T1**	**% Diff**	**T0**	**T1**	**% Diff**
CON	2.19 ± 0.54	2.0 ± 0.59	−9.07	2.00 ± 0.57	1.90 ± 0.58	−5.13	3.0 ± 0.63	2.74 ± 0.73	−9.06	2.68 ± 0.71	2.44 ± 0.71^*^^#^	−9.38
BM	2.29 ± 0.63	2.22 ± 0.61	−3.10	2.05 ± 0.63	1.96 ± 0.61	−4.49	3.04 ± 0.63	2.83 ± 0.73	−7.16	2.53 ± 0.72	2.52 ± 0.74	−0.39

*Data reported as means ± SD and percentage difference (% diff). Motivation score for classroom-based mathematics for control group (CON) and intervention group (BM). Motivation is divided into External Regulation, Introjected Regulation, Identified Regulation, and Intrinsic motivation, and were measured before (T0) and after (T1) the 6 weeks intervention period*.

* Indicates a significant difference between the same motivation factor both in different teaching approach (Basketball sessions combined with mathematics and classroom-based mathematics)

# *Indicates a significant interaction from T0 to T1 between CON and BM*.

## Discussion

### Experiences of Competence, Autonomy, and Intrinsic Motivation During Sessions of Basketball Combined With Mathematics Compared to Usual Classroom-Based Mathematics

In the present study, it is shown that combining and integrating mathematics into basketball in a school setting were associated with higher acute levels of experienced autonomy, competence, and intrinsic motivation than classroom-based mathematics within the BM group. Intrinsic motivation for a learning activity reflects that children enjoy the activities and experience more engagement due to this enjoyment (Ryan and Deci, 2000). As described in SDT, maintaining intrinsic motivation for an activity is dependent on the participants experiencing autonomy, competence, and relatedness during the activity (Deci and Ryan, 1985; Reis et al., 2000). Using mathematics in more concrete tasks, such as the basketball exercises in this present study, might have made mathematics seem more relevant to the pupils, which, in other studies, has shown to increase student's perceived autonomy (Su and Reeve, 2011), intrinsic motivation for, and involvement in the learning activities (Weaver and Cottrell, 1988; Sass, 1989; Frymier and Shulman, 1995; Simons et al., 2003; Kember et al., 2008). This is supported theoretically by the self-determination theory (Deci and Ryan, 1985) where relevance is described as important to the internalization process, promoting a more autonomous motivation for the activity. It might also be that a more concrete practical use of mathematics gives children more experiences of competence.

These findings are in line with other studies where physical activity is integrated in the learning situations (Vazou et al., 2012; Vazou and Smiley-Oyen, 2014; Damsgaard et al., 2020). Damsgaard et al. showed that integrating movement in the teaching situation increased children's learning (letter recognition) but also increased the children's intrinsic motivation (Damsgaard et al., 2020). In the present study, we have not assessed academic performance as an outcome, however, it could be relevant to investigate whether increased motivation in the BM group positively affects academic performance.

### Effect of the Intervention on Children's Motivation Compared to the Control Situation

The results from the SRQ-A questionnaire implied that having basketball combined with mathematics (BM) sessions can help maintain children's intrinsic motivation for classroom-based mathematics compared to a control situation without BM (CON).

Intrinsic motivation for a subject has shown to be of importance to academic achievement (Gottfried, 1985; Ryan and Deci, 2002; Singh et al., 2002; Ryan, 2009; Gutman et al., 2010). Furthermore, it can be argued that children's enjoyment of mathematics and of classroom activities is an important goal in itself. The potential supplementary function of why basketball combined with mathematics is motivating for mathematics, in general, is in accordance with the hierarchical nature of motivation, which proposes that motivation from one teaching situation can lead to an increase in general school motivation (Vallerand, 2000).

However, no differences were seen between the intervention group and the control group's development in extrinsic types of motivation for mathematics. It was hypothesized that having concrete experiences with the usefulness of mathematics in solving concrete tasks in BM would also increase the students' Extrinsic Motivation Identified. However, this hypothesis could not be confirmed by the data.

Furthermore, no differences were seen in the two most controlled forms of motivation, introjected and external regulation, which can be seen as a quality of the intervention, as these types of motivations often have a negative impact on long term enjoyment, engagement, and continuation with the activities (Guay et al., 2010).

### Practical Recommendations for Basketball Combined With Mathematics and Perspectives

This concept is developed in a Danish school context with the perspective of what is possible. The model might give new ideas for the development of other teaching concepts and, therefore, some reflections on the basis of basketball combined with mathematics (further details are found in Wienecke and Damsgaard, 2020). The teaching setup of having this type of teaching within the physical education classes once a week is suitable to the minimum standard of all public schools in Denmark. The duration of 6 weeks fits in between school vacations so the course can be taken in one coherent period at several different times during the school year, which makes this model practically feasible in a school setting. Facilities at schools varies but the idea behind this intervention, is that you only need two baskets pr. class and one ball for every four children. These minimalistic requirements make it easy for all public schools to use the activities. Scaling up or reconfiguration is easy both in terms of lesson frequency, intensity, and in relation to the structure of the activities.

The structure of the activities is based on some important concepts to create a good learning environment such as togetherness, play and equal focus on developing skills in both basketball and math. The children are always assigned into groups of two or more. This can create a feeling of solving the assignments together and avoid loneliness and incompetence when assignments are found difficult and hopefully, get the common joy of that. It is equally important to develop skills within basketball and as well as in math. The underlying intention is that the children should have the feeling of becoming better, for example, at scoring points and/or solving the math assignments. Implicit in the assignments is that the children have to think, reflect, talk, and use the language of math which can help the children in verbalizing the math terminology and become confident with it.

The assignments are usually starting out in the easiest way and then after the first or second round the assignments are adjusted. For example, shooting drills begin close to the basket so the children hit the basket as often as possible, and then later, the distance increases and the difficulty of hitting the basket will likewise be more challenged. This is the same for the math part (i.e., easy start and then more difficult).

Competition is an element in basketball combined with mathematics but never as a traditional five on five game. The children often compete indirectly. Indirect competition could, for example, be reflected in a situation where the children have to finish first within an assignment but during the competition, they do not always know how far the others are. This practical recommendation can facilitate that the children focus on their own performance instead of on the competitors. The play element is very important. The children should have the feeling of being included in the task and also have the possibility to solve the assignments in their own way or develop their own ideas within the frame of the lesson.

The finding that sessions with basketball combined with mathematics (BM) has a positive effect on children's general intrinsic motivation for classroom-based mathematics is remarkable and supports initiatives where integrating physically active games and play with curriculum based academic tasks are used as a supplement to the more traditional classroom teaching. In this study, the combination of basketball and mathematics was chosen, but physically active play-based teaching methods could be combined with other theoretical themes such as language, biology, physics, etc.

For further exploration of the effects of basketball sessions combined with mathematics, it would be interesting to investigate both short- and long-term effects on the children's mathematical performance before and after a BM intervention.

### Strengths and Limitations of the Study

The present study is unique in its study design and sample size. The intervention intensity (i.e., once a week of basketball combined with mathematics) can easily be organized in the public school because it only requires the allocation of physical education classes. The BM does not require an extensive teaching material but only two baskets and some basketballs. As described above, only a few studies have investigated the effects of integrating physical activity and academic learning in a meaningful way as in this study. A limitation of the study is that only a few acute motivation measures were collected (see Figure 2). Furthermore, the intervention group, BM, had external trained researchers to carry through the project, which may have led to differences in the teaching capacity and learning environment. Due to the large number of school classes, trained researchers and teachers involved in this present study the bias should be small. Furthermore, the analysis was adjusted for clustering on school and grade level. Finally, it could have been interesting to measure the children's academic achievement, however, this was beyond the purpose of this study.

## Conclusions

This study shows that basketball combined with mathematics is an intrinsically motivating way to practice mathematics, which also has a positive influence on children's more general intrinsic motivation for mathematics in the classroom.

## Data Availability Statement

The raw data supporting the conclusions of this article will be made available by the authors, without undue reservation.

## Ethics Statement

The studies involving human participants were reviewed and approved by the Local Ethical Committee at UCPH. Written informed consent to participate in this study was provided by the participants legal guardian/next of kin.

## Author Contributions

JW, JH, and GN designed the experiment. JH and KM collected the data. LD conducted the required data analysis and the first draft of the manuscript. All authors contributed to drafting the manuscript and approved the final version of the manuscript.

## Conflict of Interest

The authors declare that the research was conducted in the absence of any commercial or financial relationships that could be construed as a potential conflict of interest.
