# Supplementary material for: Six Weeks of Basketball Combined With Mathematics in Physical Education Classes Can Improve Children's Motivation for Mathematics
Source: Front Psychol. 2021 Mar 26;12:636578. doi: 10.3389/fpsyg.2021.636578 (PMC8034423; doi:10.3389/fpsyg.2021.636578)
Supplement: Supplementary file 1 [file Table_1.pdf]

Appendix 1. Factor Loadings of Items of IMI Questionnaire

	Loadings IMI BM			Loadings IMI CM		
Question:	Intrinsic Motivation	Autonomy	Competence	Intrinsic Motivation	Autonomy	Competence
**IM1**	**0.862**	0.026	0.098	**0.927**	0.012	0.012
**IM2**	**0.983**			**0.922**		0.043
**IM3**	**0.363**	0.301	0.005	**0.637**	0.132	
**IM4**	**0.502**	0.131	0.169	**0.591**	0.147	0.113
**AUT1**	0.339	**0.517**		0.317	**0.492**	
**AUT2**	0.328	**0.459**	0.188	0.319	**0.334**	0.287
**AUT3**		**0.788**			**0.76**	
**COM1**		0.003	**0.961**			**0.932**
**COM2**	0.12		**0.733**	0.048	0.048	**0.721**
**COM3**		0.216	**0.335**		0.24	**0.291**
**Cronbach's Alpha of Intended Items**	0.777	0.603	0.627	0.847	0.618	0.67

Appendix 1. Factor loadings of the Acute Intrinsic Motivation IMI questionnaire within the intervention group having Basketball combined with mathematics (BM) and when having classroom-based mathematics (CM). Bold indicates loadings on intended factor. Cronbach's Alpha of intended items are represented.
