# Supplementary material for: Six Weeks of Basketball Combined With Mathematics in Physical Education Classes Can Improve Children's Motivation for Mathematics
Source: Front Psychol. 2021 Mar 26;12:636578. doi: 10.3389/fpsyg.2021.636578 (PMC8034423; doi:10.3389/fpsyg.2021.636578)
Supplement: Supplementary file 2 [file Table_2.pdf]

Appendix 2. Factor Loadings of Items of SRQ-A

	Loadings SRQ-A			
**Question:**	**Intrinsic Motivation**	**Identified**	**Introjected**	**External**
**IM1**	**0.585**	0.254	0.176	
**IM2**	**0.653**	0.267	0.147	
**IM3**	**0.797**	0.025		0.061
**IM4**	**0.801**	0.054		0.154
**IM5**	**0.425**	0.446	0.098	0.069
**Identified1**	0.357	**0.444**	0.089	
**Identified2**	0.285	**0.504**	0.115	0.098
**Identified3**	0.326	**0.084**	0.032	0.17
**Identified4**	0.296	**0.399**	0.083	0.255
**Identified5**	0.156	**0.621**	0.165	0.276
**Introjected1**	0.226		**0.568**	0.214
**Introjected2**		0.275	**0.603**	
**Introjected3**		0.309	**0.682**	0.044
**Introjected4**	0.12		**0.566**	0.242
**Introjected5**	0.193	0.312	**0.441**	
**Introjected6**	0.322		**0.632**	0.209
**Introjected7**		0.381	**0.523**	0.074
**External1**			0.487	**0.306**
**External2**		0.196		**0.644**
**External3**		0.247		**0.757**

**External4**	0.245		0.603	**0.25**
**External5**			0.455	**0.346**
**External6**		0.113		**0.759**
**External7**	0.211		0.293	**0.164**
**Cronbach's Alpha of Intended Items**	0.804	0.696	0.761	0.696

Appendix 2. Factor loadings of the Acute Intrinsic Motivation IMI questionnaire within the intervention group having basketball combined with mathematics (BM) and when having classroom-based mathematics (CM). Bold indicates loadings on intended factor. Cronbach's Alpha of intended items are represented.
